# COVID-19 - The clinical consequences of social isolation and the relation with sleep bruxism and comorbidities

**DOI:** 10.5935/1984-0063.20210004

**Published:** 2021

**Authors:** Thays Crosara Abrahão Cunha, Thulio Marquez Cunha, Abadia Gilda Buso Matoso, Eduardo Januzzi, Cibele Dal-Fabbro

**Affiliations:** 1 Federal University of Uberlândia, Institute of Biotechnology - Uberlandia - Minas Gerais - Brazil.; 2 Federal University of Uberlândia, Department of Pneumology -Uberlandia - Minas Gerais - Brazil.; 3 Federal University of Uberlândia, Department of Clinical Medicine -Uberlandia - Minas Gerais - Brazil.; 4 Ciodonto, Postgraduate Program in Oral Facial Pain - Belo Horizonte - Minas Gerais - Brazil.; 5 Hospital Mater Dei, Oral Facial Pain Center - Belo Horizonte - Minas Gerais - Brazil.; 6 Federal University of São Paulo, Sleep Institute - São Paulo - São Paulo - Brazil.; 7 Universite de Montreal, Center for Advanced Research in Sleep Medicine - Montreal - Canada.

**Keywords:** Gastroesophageal reflux, Pandemic, Sleep bruxism, Anxiety obesity

## Abstract

SARS-COV-2 is a highly pathogenic coronavirus that causes the disease known as COVID-19, which has infected more than 100 million people worldwide. The main form of containment of the pandemic is social isolation. However the isolation, the severity of the COVID-19 disease, the uncertainty of the future and the economic impact are the possible causes of anxiety as an adverse effect of the pandemic. The literature describes the possible association between anxiety with poor sleep quality, exacerbation of painful conditions, gastroesophageal reflux disease, increased consumption of drugs and the possibility of developing or enhancing sleep bruxism. Health professionals should keep in mind the possibility of overlapping with the different clinical conditions mentioned and the need for a multi-professional team to manage these patients.

SARS-CoV-2 is a highly pathogenic coronavirus that causes the disease known as COVID-19. Its pattern of lethality, mortality, infectivity, and transmissibility is not yet established. Vaccines are not yet available for the entire population, there are no specific drugs available and treatment is supportive and non-specific^1^. To date, more than 100 million people have contracted COVID-19, of which more than 2 million have died worldwide^2^. The main form of containment of the pandemic is social isolation^3^. The state of tension, thoughts of concern associated with changes in routine and lifestyle favor the manifestation of signs and symptoms of anxiety, negatively impacting even more the population’s general quality of life and health^3, 4^ (Figure 1).

**Figure 1:**
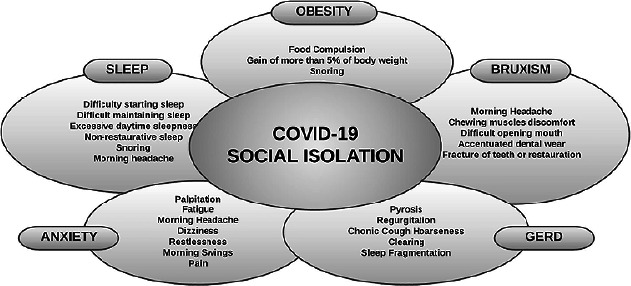
The clinical consequences of social isolation and the relation with sleep bruxism and comorbidities.

Studies have shown that social isolation, lifestyle change, the severity of the COVID-19 disease, concern for family members, the uncertainty of the future and the economic impact are the possible causes of anxiety as an adverse effect of the pandemic^3, 4^. The main symptoms are palpitation, fatigue, headache, dizziness, restlessness, mood swings, feelings of fear, difficulty in concentration, difficulty in maintaining a good quality of sleep, and an even greater tendency towards isolation^3^. The individuals most likely to develop the above symptoms are those who contracted COVID-19 and their families, those who already had some physical or psychiatric morbidity and health professionals^4^. As a preventive way, we must alert the population about the risks of psychosocial changes, the possible physical and metabolic consequences, and motivate them to adopt strategies to prevent psychological disease and the importance of health promotion.

Social isolation has a great influence on the pace of the population’s life. Changing daily routines and changing habits compromise sleep quality. This fact is mainly due to the possibility of naps, changes in time and total sleep time, and also by the stress conditions imposed by the pandemic^5^. The relationship between anxiety and poor sleep quality is bidirectional^5^. Insomnia and/or lack of sleep are common symptoms in individuals with anxiety disorders, while acute sleep deprivation is considered an anxiogenic factor^5^. The introduction of sleep hygiene, aimed at maintaining the duration and adequate quality of sleep can lead to an increase in quality of life. These measures directly prevent sleep-related disorders, and indirectly, reduce anxiety-related comorbidities^5^.

Social isolation can also lead some individuals to have more pain in response to stress, due to many different reasons. Complaints of headache and myofascial pain before the isolation can be worsened due to anxiety^5^. Pain can directly interfere on sleep quality, as well as a bad night of sleep can worsen the pain or even cause it. The relationship between acute pain and sleep is more linear (in the presence of pain, sleep tends to be worse)^6^. However, the relationship between sleep disorders and chronic pain is circular and bidirectional, in a way that one is worsening the other and both managements should be encouraged to achieve a better quality of life^6^.

The relationship between stress and gastroesophageal reflux disease (GERD) is also considered bidirectional^7^. GERD is characterized by a retrograde flow of gastrointestinal content towards the esophagus and adjacent organs and may manifest itself by typical symptoms (heartburn and regurgitation) and/ or atypical symptoms (chronic cough, hoarseness, throat clearing, and sleep disturbances)^7^. Anxiety can reduce the tone of the lower esophageal sphincter, increase the number of ineffective esophageal contractions, and enhance the permeability of the gastric mucosa, generating a greater propensity for the development of peptic esophagitis and esophageal hypersensitivity^7^. It is important to consider that in addition to factors related to anxiety, the conditions inherent to the pandemic and social isolation can favor obesity and, as a consequence, also potentiate GERD, such as: increased consumption of food and less healthy foods, a greater intake of alcoholic beverages and psychotropic drugs, and the lower frequency of physical activities.

The literature also describes a higher latency for sleep, a higher rate of awakenings and a higher incidence of sleep bruxism (BS) in patients with GERD. These clinical conditions occur with severity association. A higher degree of anxiety is associated with more severe GERD’s signs and symptoms and more frequent symptoms of insomnia and bruxism^7, 8^.

Isolation conditions lead to less food availability and greater difficulty in acquiring fresh food, favoring less healthy eating, making evident the trend towards a more caloric diet, with more carbohydrates and fat. In addition, anxiety can generate the need to eat in compensation to “feel better”. This is also the explanation for the greater intake of alcoholic beverages, cigarettes, illicit drugs, and psychotropic drugs in periods of social isolation. The closure of parks, squares, and gyms also causes isolated people to greatly reduce their usual physical activities^8^. All of these factors favor weight gain and increase the risks of obesity.

Obesity also has an important interface regarding both to sleep quality, OSA (obstructive sleep apnea) and GERD^9, 10^. During sleep a series of hormones are secreted, among them leptin and ghrelin, known as hormones of satiety and hunger, respectively^9^. Studies describe that leptin is decreased and ghrelin increased in sleep deprived individuals^9^. For the shortest sleep time and the longest waking period, sleep-deprived individuals would have more time available to eat and less willingness to perform physical activity^9^. All of these conditions would favor weight gain and consequently obesity.

On the other hand, the increased BMI is also considered a risk factor for GERD^10^. Obese individuals have increased intra-abdominal pressure and transient relaxation of the lower esophageal sphincter, greater risk of hiatal hernia, and less gastric emptying, favoring the retrograde flow of stomach contents^10^. Therefore, individuals who already have an increased BMI or those who have increased their body weight by 5% during the isolation period, should be aware of the quality of sleep and the signs and symptoms of anxiety^10^.

Bruxism can occur during sleep and/or wakefulness^11^. It is characterized by increased masticatory muscle activity, has a multifactorial etiology and is modulated by the central nervous system^11^. The literature describes the association of both bruxism with anxiety and GERD, with poor quality sleep and indirectly with obesity, as described below^11^.

Psychosocial changes are considered a possible etiological factor of SB^11^. Poor sleep quality and insomnia signs and symptoms have been associated with both anxiety disorders and sleep bruxism^12, 13, 14^. The individuals more sensitive to stress, who need a greater sense of security, those with panic symptoms and anxiety profile are more prone to the development of bruxism^15^. It is also important to consider that bruxism can be triggered or worsened as a side effect of some medications used to control anxiety and depression symptoms, such as serotonin reuptake inhibitors^16^. Patients who report a complaint of SB should be asked about psychosocial changes and should be indicated to cognitive behavioral therapy, relaxation therapies, and investigation of the possibility of changing medications, when appropriate^11, 12, 13, 14, 15, 16^.

Regarding GERD, SB is considered a possible protective factor for the disease^11, 14, 17, 18^. This hypothesis describes that interdental contact from SB would activate the mechanoreceptors of the periodontal ligament, stimulating salivary secretion and neutralizing the acidic pH of the oral cavity^11, 14, 17, 18^. Polysomnographic studies that evaluated patients with sleep bruxism and GERD support this hypothesis^11, 14, 17, 18^. More studies need to be carried out to establish and understand this relationship.

Indirectly, SB may also be associated with obesity. One of the main clinical predictors of OSA is increased BMI. It is a chronic disease, characterized by obstruction of the upper airways while the patient sleeps, leading to sleep fragmentation, and a state of chronic sleep deprivation^12^. The literature does not establish a cause and effect relationship between OSA and SB events, but supports the hypothesis that the two clinical conditions overlap^12, 19^. Patients who report signs and symptoms of bruxism after weight gain should be investigated for obstructive sleep apnea^14^. The main clinical signs of this disease are snoring, excessive daytime sleepiness, and non-restorative sleep^12^.

As we can observe, the social isolation and stress conditions imposed by the current pandemic, besides compromising the quality of life and generating risks to the general health of the population, can also trigger or potentiate the events of SB. The establishment of a routine, the maintenance of regular sleep schedules, the adoption of controlled and balanced nutrition, and the regular practice of physical activity, should be recommended in order to minimize the adverse effects of social isolation^20^.

Health professionals should keep in mind the possibility of overlapping with the different clinical conditions mentioned and the need for a multi-professional team to manage these patients. The signs and symptoms of anxiety should be better analyzed by psychologists and/or psychiatrists. In cases of weight gain, support with a nutritionist and endocrinologist is recommended. GERD must be monitored and managed by gastroenterologists, while bruxism must be evaluated and controlled by dental surgeons trained in sleep dentistry and/ or temporomandibular disorders and orofacial pain. Detailed, individualized and comprehensive anamnesis is essential for establishing the correct diagnosis and defining the therapeutic and/or supportive conduct for these patients.
